# Epstein-Barr virus as promoter of Lemierre syndrome: systematic literature review

**DOI:** 10.1007/s00405-024-08767-x

**Published:** 2024-06-05

**Authors:** Alessia A. Delcò, Sara M. M. A. Montorfani, Renato Gualtieri, Sebastiano A. G. Lava, Gregorio P. Milani, Mario G. Bianchetti, Gabriel Bronz, Pietro B. Faré, Lisa Kottanattu

**Affiliations:** 1 Family Medicine, Faculty of Biomedical Sciences, Università Della Svizzera Italiana, Lugano, Switzerland; 2 Pediatric Cardiology Unit, Department of Pediatrics, Centre Hospitalier Universitaire Vaudois and University of Lausanne, Lausanne, Switzerland; 3 Clinical Pharmacology Service, Centre Hospitalier Universitaire Vaudois and University of Lausanne, Lausanne, Switzerland; 4 Pediatric Unit, Fondazione IRCCS Ca’ Granda Ospedale Maggiore Policlinico, Via Della Commenda 9, 20122 Milan, Italy; 5 Department of Clinical Sciences and Community Health, Università Degli Studi Di Milano, Milan, Italy; 6 Department of Infectious Diseases and Hospital Epidemiology, University Hospital Zurich, Zurich, Switzerland; 7 Pediatric Institute of Southern Switzerland, Ente Ospedaliero Cantonale, Bellinzona, Switzerland; 8 Faculty of Biomedical Sciences, Università Della Svizzera Italiana, Lugano, Switzerland

**Keywords:** Epstein-Barr virus, Human herpesvirus 4, Lemierre syndrome, Necrobacillosis, Postanginal sepsis

## Abstract

**Purpose:**

To investigate a possible link between acute Epstein-Barr virus infection and Lemierre syndrome, a rare yet life-threatening infection.

**Methods:**

A systematic review was conducted adhering to the 2020 Preferred Reporting Items for Systematic Reviews and Meta-Analyses guidelines. Diagnosis criteria for Lemierre syndrome were established, and data extraction encompassed demographic data, clinical, and laboratory information.

**Results:**

Out of 985 initially identified papers, 132 articles were selected for the final analysis. They reported on 151 cases of Lemierre syndrome (76 female and 75 male patients with a median of 18 years) alongside interpretable results for Epstein-Barr virus serology. Among these, 38 cases (25%) tested positive for acute Epstein-Barr virus serology. There were no differences in terms of age, sex, or Fusobacterium presence between the serologically positive and negative groups. Conversely, instances of cervical thrombophlebitis and pulmonary complications were significantly higher (P = 0.0001) among those testing negative. The disease course was lethal in one case for each of the two groups.

**Conclusions:**

This analysis provides evidence of an association between acute Epstein-Barr virus infection and Lemierre syndrome. Raising awareness of this link within the medical community is desirable.

**Supplementary Information:**

The online version contains supplementary material available at 10.1007/s00405-024-08767-x.

## Introduction

Lemierre syndrome, also known as postanginal sepsis or necrobacillosis, is an infrequent yet potentially fatal infection, that usually affects immunocompetent individuals. It is characterized by an acute oropharyngeal inflammation, which is followed by a septic cervical (mostly jugular) thrombophlebitis, which, in turn, leads to the dissemination of septic emboli [1–4]. Fusobacterium species, part of the oral microbiota, are the primary causative agents [1–4]. The condition was initially documented in 1900 by Paul Courmont [5], followed by Mark S. Reuben in 1936 [6]. However, André Lemierre in France provided the most detailed description in 1936 [7].

Viral agents may compromise mucous membrane integrity, providing an entry point for bacterial pathogens and increasing the susceptibility to various invasive infections, including those caused by meningococci [8, 9]. A link between acute infection caused by the human herpes virus 4, also known as Epstein-Barr virus [10, 11], and Lemierre syndrome has been suggested [3]. This report aims to systematically explore this association.

## Methods

This systematic review (registered on INPALSY, number 202410102) adhered to the 2020 edition of the Preferred Reporting Items for Systematic Reviews and Meta-Analyses (PRISMA) guidelines [12]. The data were sourced from Web of Science, the United States National Library of Medicine, and Excerpta Medica. The search strategy focused on the term "Lemierre syndrome" across the three databases. Additionally, articles identified in the references of retrieved records, reports available in Google Scholar, and articles already familiar to the authors were included [13]. The searches were conducted in July 2023 and repeated prior to submission (February 28, 2024).

Eligible were reports of apparently immunocompetent patients with a diagnosis of Lemierre syndrome and with either a positive or negative serology for Epstein-Barr virus.

Since a formal case definition for Lemierre syndrome has not yet been established [1–4], we established this diagnosis in patients with an acute onset pharyngeal inflammation associated with (a) isolation of Fusobacterium species from a blood culture or a normally sterile site, or (b) a cervical thrombophlebitis associated with one of the following features: pulmonary involvement (infiltrates, septic emboli, abscesses, or empyema; an isolated pleural effusion was not considered sufficient to define lung impairment); metastatic extra-pulmonary involvement such as abscesses or septic emboli; or isolation of a germ other than Fusobacterium from a blood culture or a normally sterile site. Cases of Lemierre syndrome temporally associated with a urogenital infection, or surgery were excluded. Cases related to a significant odontogenic infection were also not included [14]. A local spread of the oropharyngeal infection was not regarded as systemic involvement in the diagnosis of Lemierre syndrome.

Patients with a positive Paul-Bunnell-Davidsohn heterophile test, IgM and IgG antibodies to the Epstein-Barr viral capsid, or IgG antibodies to the early Epstein-Barr viral antigen were deemed to have a positive serology for acute Epstein-Barr Virus infection [10, 11]. Conversely, the serology for acute Epstein-Barr Virus infection was considered negative in cases with isolated IgG antibodies to the Epstein-Barr viral capsid; negativity for IgM antibodies to the Epstein-Barr viral capsid; positivity for IgG to Epstein-Barr virus nuclear antigen; or negative Paul-Bunnell-Davidsohn test [10, 11]. Cases that were reported as serologically positive respectively negative for an acute Epstein-Barr virus infection but lacked information regarding the performed serological tests were also included. For both Epstein-Barr virus positive and negative cases, demographic details, clinical and laboratory data, and outcomes were collected.

Two authors in duplicate conducted the literature search, selected eligible studies, extracted data, and assessed the comprehensiveness of each included case. Disagreements were resolved through discussions, involving a senior author if needed. One author inputted data into a worksheet, and the second author verified data accuracy.

The omnibus normality test disclosed that continuous variables were not normally distributed [15]. Hence, the latter are presented as median and interquartile range, and their analysis was conducted using the Mann–Whitney-Wilcoxon test for two independent samples [16]. Categorical variables are expressed as counts and were analyzed by means of the Fisher exact test [16]. A significance level was assigned at < 0.05 for a two-sided P-value.

## Results

**Fig. 1:**
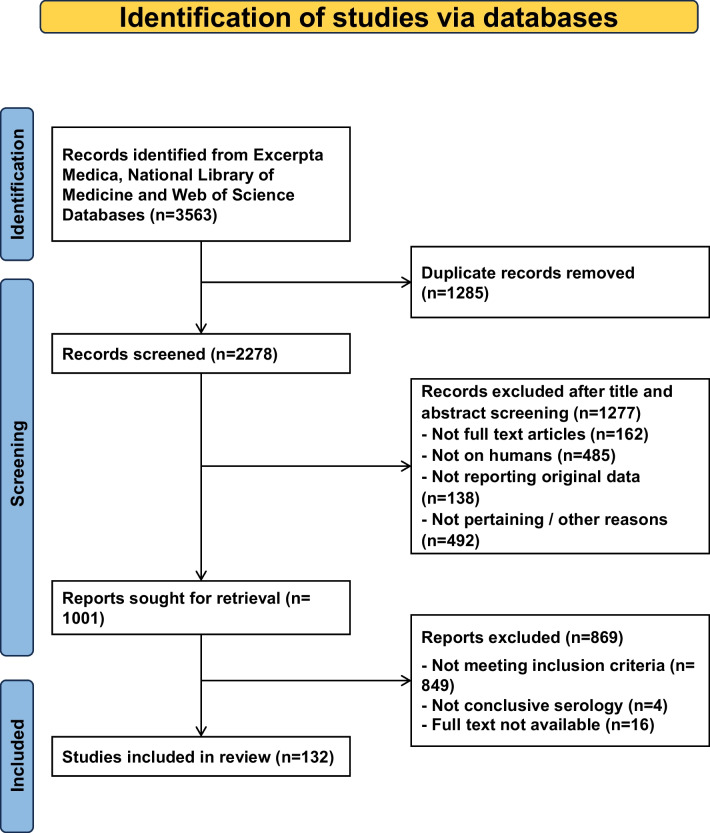
Epstein-Barr virus as promoter of Lemierre syndrome. Flowchart of the literature search

The literature search process is outlined in Fig. 1. The full-text of 1001 papers was assessed. For the final analysis, we included 132 articles [see: supplementary document] published after 1979: 70 from America (United States of America, N = 64; Canada, N = 5; Jamaica, N = 1), 53 from Europe (United Kingdom, N = 15; Germany, N = 6; France, N = 5; Greece, N = 5; Spain, N = 4; Denmark, N = 3; Netherlands, N = 3; Belgium, N = 2; Italy, N = 2; Portugal, N = 2; Sweden, N = 2; Switzerland, N = 2; Austria, N = 1; Norway, N = 1) and 9 from Asia (Türkiye, N = 3; Israel, N = 2; Japan, N = 2; Pakistan, N = 1; Sri Lanka, N = 1). One hundred twenty-two articles were written in English, three in French, three in German, two in Spanish, and each one in Norwegian and Swedish. The mentioned 132 articles [15–146] described subjects with a Lemierre syndrome and an interpretable serology for Epstein-Barr virus.

The mentioned reports provided information about 151 cases of Lemierre syndrome (76 female and 75 male individuals 18 [16–23] years of age) with an interpretable serology for Epstein-Barr virus infection (Table 1). The acute Epstein-Barr virus serology was positive in 38 (25%) and negative in 113 (75%) cases. Cases with and without serological evidence of acute Epstein-Barr virus infection did not significantly differ with respect to female-male-ratio, age, positivity for *Fusobacterium species *or extrapulmonary involvement. A cervical thrombophlebitis (75% versus 39%) and a pulmonary involvement (87% versus 55%) were more frequently (P = 0.0001) observed in cases with a negative acute Epstein-Barr virus serology. The disease course was lethal in one case for each of the two groups.

**Table 1:** Clinical features in 151 patients (ranging in age from 3.5 to 70 years) affected by Lemierre syndrome with and without laboratory features consistent with an acute Epstein-Barr virus infection

	All cases	Serology for Acute Epstein-Barr Virus Infection		P–values^&^
		Positive	Negative	
N (%)	151 (100)	38 (25)	113 (75)	
Demographics				
Females: males, N (%)	76 (50): 75 (50)	23 (61): 15 (39)	53 (47): 60 (53)	0.1894
Age, years	18 [16–23]	19 [17–21]	18 [16–23]	0.7864
Fusobacterium species positivity, N (%)	121 (80)	33 (87)	88 (78)	0.3468
Cervical vessel thrombophlebitis, N (%)	100 (66)	15 (39)	85 (75)	**0.0001**
Pulmonary involvement, N (%)	119 (79)	21 (55)	98 (87)	**0.0001**
Pneumonia, N (%)	91 (60)	12 (32)	79 (70)	**0.0001**
Abscess, N (%)	46 (30)	7 (18)	39 (36)	0.0694
Septic emboli, N (%)	44 (29)	7 (18)	37 (33)	0.1031
Empyema, N (%)	6 (4.0)	2 (5.3)	4 (3.5)	0.6417
Extrapulmonary involvement, N (%)	41 (27)	12 (32)	29 (26)	0.5290
Central nervous system, N (%)	16 (11)	6 (16)	10 (8.8)	0.2345
Musculoskeletal system, N (%)	16 (11)	6 (16)	10 (8.8)	0.2345
Hepatobiliary system, N (%)	10 (6.6)	2 (5.3)	8 (7.1)	> 0.9999
Further systems, N (%)**	7 (4.6)	2 (5.3)	5 (4.4)	> 0.9999
Death, N (%)	2 (1.3)	1 (2.6)	1 (0.9)	0.4412

Data are presented either as frequency (with percentage) or as median (with interquartile range)

^**^splenic abscess (N = 3), endocarditis (N = 3), renal abscess (N = 1), skin emboli (N = 1). ^**&**^Significant p-values are in bold

**Fig. 2:**
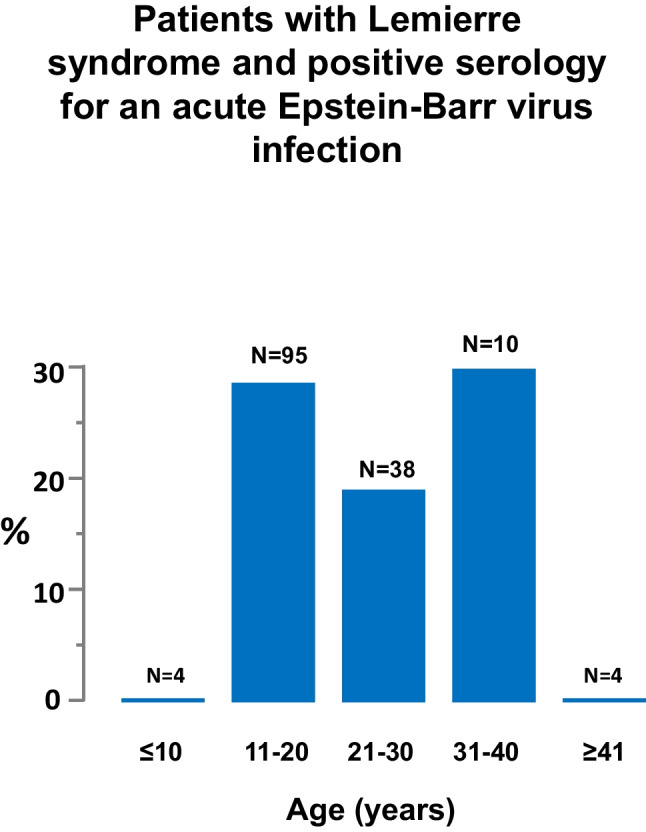
Age distribution of patients with Lemierre syndrome and positive serology for an acute Epstein-Barr virus infection

The serology for acute Epstein-Barr virus infection was never positive in individuals ≤ 10 and ≥ 41 years of age (Fig. 2).

## Discussion

Lemierre syndrome [1–4] and Epstein-Barr virus [10, 11] infectious mononucleosis predominantly occur in otherwise healthy teenagers and young adults. A link between Lemierre syndrome and serological evidence of acute Epstein-Barr virus infection was first proposed in the eighties of last century [3]. In this analysis of the literature, we identified a positive serology for an acute Epstein-Barr virus infection in 38 (25%) out of 151 patients diagnosed with Lemierre syndrome. Hence, these data allow to infer that Epstein-Barr virus infection may sporadically predispose individuals to develop Lemierre syndrome.

At least two mechanisms might underly the link between Epstein-Barr virus infection and Lemierre syndrome. Firstly, there is a higher prevalence of *Fusobacterium* positivity in individuals with infectious mononucleosis as opposed to those who are healthy [17]. Furthermore, in instances where the Fusobacterium swab yields positive results, the bacterial load is elevated in patients with infectious mononucleosis [17]. Secondly, the infiltration of bacteria into the tonsillar epithelium is increased in individuals with Epstein-Barr virus infectious mononucleosis [18].

Cervical thrombophlebitis and pulmonary involvement occurred more frequently in instances where there was a negative acute Epstein-Barr virus serology. The reasons behind this observation remain unexplained.

This analysis exhibits both limitations and strengths. The main weakness is the limited dataset: only 151 instances of Lemierre syndrome with associated Epstein-Barr virus serology were detected, highlighting the need for broader, prospective research. However, the rarity of this condition, evidenced by a Danish report of an incidence rate of 3.6 per million annually [19], complicates such research efforts. In contrast, the study’s strengths include adherence to established methodologies and the comprehensive analysis of data from three distinct databases.

## Conclusion

This literature review, taken together with experimental data [127, 18], support the link between Epstein-Barr virus infectious mononucleosis and Lemierre syndrome, highlighting the importance of increasing awareness within the medical community. Even though it is rare, healthcare providers should keep Lemierre syndrome in mind when infectious mononucleosis patients acutely present with high fever, deterioration of general well-being, unilateral neck pain, or shortness of breath.

## Supplementary Information

Below is the link to the electronic supplementary material.

Supplementary file1 (DOCX 38 KB)

## Data Availability

Data sharing is not applicable to this article as no new data were generated in this study.
