# Supplementary material for: Epstein-Barr virus as promoter of Lemierre syndrome: systematic literature review
Source: Eur Arch Otorhinolaryngol. 2024 Jun 5;281(10):5497–502. doi: 10.1007/s00405-024-08767-x (PMC11416426; doi:10.1007/s00405-024-08767-x)
Supplement: Supplementary file 1 — Supplementary file1 (DOCX 38 KB) [file 405_2024_8767_MOESM1_ESM.docx]

**Articles included for the final analysis**

1. Vogel LC, Boyer KM (1980) Metastatic complications of Fusobacterium necrophorum sepsis. Two cases of Lemierre's postanginal septicemia. Am J Dis Child 134(4):356-358. https://doi.org/10.1001/archpedi.1980.04490010014005

2. Adams J, Capistrant T, Crossley K, Johanssen R, Liston S (1983) Fusobacterium necrophorum septicemia. JAMA 250(1):35. https://doi.org/10.1001/jama.1983.03340010020018

3. Dagan R, Powell KR (1987) Postanginal sepsis following infectious mononucleosis. Arch Intern Med 147(9):1581-1583. https://doi.org/10.1001/archinte.1987.00370090059012

4. Gruber B, Mhoon EE (1987) Bilateral deep space neck abscesses complicating infectious mononucleosis. Otolaryngol Head Neck Surg 97(1):66-68. https://doi.org/10.1177/019459988709700112

5. Moreno S, García Altozano J, Pinilla B, López JC, de Quirós B, Ortega A, Bouza E (1989) Lemierre's disease: postanginal bacteremia and pulmonary involvement caused by Fusobacterium necrophorum. Rev Infect Dis 11(2):319-324. https://doi.org/10.1093/clinids/11.2.319

6. Sinave CP, Hardy GJ, Fardy PW (1989) The Lemierre syndrome: suppurative thrombophlebitis of the internal jugular vein secondary to oropharyngeal infection. Medicine (Baltimore) 68(2):85-94.

7. Rathore MH, Barton LL, Dunkle LM (1990) The spectrum of fusobacterial infections in children. Pediatr Infect Dis J 9(7):505-508. https://doi.org/10.1097/00006454-199007000-00010

8. Cosgrove EF, Colodny SM, Pesce RR (1993) Adult respiratory distress syndrome as a complication of postanginal sepsis. Chest 103(5):1628-1629. https://doi.org/10.1378/chest.103.5.1628

9. Ieven M, Vael K, De Mayer M, De Schepper A, Pattyn S (1993) Three cases of Fusobacterium necrophorum septicemia. Eur J Clin Microbiol Infect Dis 12(9):705-706. https://doi.org/10.1007/BF02009385

10. Mann KA (1994) Lemierre’s syndrome following infectious mononucleosis. Clin Microbiol Newsl 16:158–159.

11. Alvarez A, Schreiber JR (1995) Lemierre's syndrome in adolescent children - anaerobic sepsis with internal jugular vein thrombophlebitis following pharyngitis. Pediatrics 96(2 Pt 1):354-359. https://doi.org/10.1542/peds.96.2.354

12. Gold WL, Kapral MK, Witmer MR, Mahon WA, Ostrowski M, Vellend H (1995) Postanginal septicemia as a life-threatening complication of infectious mononucleosis. Clin Infect Dis 20(5):1439-1440. https://doi.org/10.1093/clinids/20.5.1439

13. Gupta M, Castello FV, Kesarwala HH (1995) Respiratory failure caused by Lemierre's syndrome. Clin Pediatr (Phila) 34(5):275-277. https://doi.org/10.1177/000992289503400510

14. Koay CB, Heyworth T, Burden P (1995) Lemierre syndrome - a forgotten complication of acute tonsillitis. J Laryngol Otol 109(7):657-661. https://doi.org/10.1017/s0022215100130956

15. Martin MJ, Wright ED (1995) A case of Fusobacterium necrophorum sepsis. J Infect 31(2):151-152. https://doi.org/10.1016/s0163-4453(95)92235-0

16. Busch N, Mertens PR, Schönfelder T, Nguyen H, Marschall HU, Kierdorf H, Haase G, Urhahn R, Sieberth HG, Matern S (1996) Postangina-Sepsis Lemierre mit Meningitis und intravasaler Verbrauchskoagulopathie als Komplikation einer infektiösen Mononukleose mit Pansinusitis [Lemierre's post-tonsillitis sepsis with meningitis and intravascular consumption coagulopathy as complication of infectious mononucleosis with pansinusitis]. Dtsch Med Wochenschr 121(4):94-98. https://doi.org/10.1055/s-2008-1042978

17. De Sena S, Rosenfeld DL, Santos S, Keller I (1996) Jugular thrombophlebitis complicating bacterial pharyngitis (Lemierre's syndrome). Pediatr Radiol 26(2):141-144. https://doi.org/10.1007/BF01372094

18. Harar RP, MacDonald A, Pullen D, Ganesan S, Prior AJ (1996) Lemierre's syndrome: are we underdiagnosing this life-threatening infection? ORL J Otorhinolaryngol Relat Spec 58(3):178-181. https://doi.org/10.1159/000276822.

19. Stahlman GC, DeBoer DK, Green NE (1996) Fusobacterium osteomyelitis and pyarthrosis: a classic case of Lemierre's syndrome. J Pediatr Orthop 16(4):529-532. https://doi.org/10.1097/00004694-199607000-00022

20. Møller K, Dreijer B (1997) Post-anginal sepsis (Lemierre's disease): a persistent challenge. Presentation of 4 cases. Scand J Infect Dis 29(2):191-194. https://doi.org/10.3109/00365549709035883

21. Vandenberg SJ, Hartig GK (1998) Lemierre's syndrome. Otolaryngol Head Neck Surg 119(5):516-518. https://doi.org/10.1016/S0194-5998(98)70114-7

22. Smith SA (1999) Respiratory failure as a complication of pharyngitis: Lemierre's syndrome. Pediatr Emerg Care 15(6):402-403. https://doi.org/10.1097/00006565-199912000-00007

23. Gowan RT, Mehran RJ, Cardinal P, Jones G (2000) Thoracic complications of Lemierre syndrome. Can Respir J 7(6):481-485. https://doi.org/10.1155/2000/451860

24. Abele-Horn M, Emmerling P, Mann JF (2001) Lemierre's syndrome with spondylitis and pulmonary and gluteal abscesses associated with Mycoplasma pneumoniae pneumonia. Eur J Clin Microbiol Infect Dis 20(4):263-266. https://doi.org/10.1007/pl00011262

25. Maalikjy Akkawi N, Borroni B, Magoni M, Vignolo LA, Padovani A (2001) Lemierre's syndrome complicated by carotid thrombosis. Neurol Sci 22(5):403-404. https://doi.org/10.1007/s100720100073

26. Laguía M, Lahoz T, Martínez J, Valero J, Fraile J, Cámara F (2001) Síndrome de Lemierre: tromboflebitis séptica de la yugular interna secundaria a amigdalitis aguda [Lemierre's syndrome: septic thrombophlebitis of the internal jugular vein secondary to acute amygdalitis]. Acta Otorrinolaringol Esp 52(2):163-166. https://doi.org/10.1016/s0001-6519(01)78193-9

27. Narsinghani U, Schmidt MB, Jacobs RF, Anand KS. (2001) Radiological case of the month: Lemierre syndrome. Arch Pediatr Adolesc Med. ;155(8):965-966. https://doi.org/10.1001/archpedi.155.8.965

28. Andrianakis IA, Kotanidou AN, Pitaridis MT, Saroglou GJ, Exarhos DN, Roussos CS, Bellenis IP (2002) Life-threatening bilateral empyema and mediastinitis complicating infectious mononucleosis. Intensive Care Med 28(5):663-664. https://doi.org/10.1007/s00134-002-1270-1

29. Hoehn S, Dominguez TE (2002) Lemierre's syndrome: an unusual cause of sepsis and abdominal pain. Crit Care Med 30(7):1644-1647. https://doi.org/10.1097/00003246-200207000-00040

30. Moore BA, Dekle C, Werkhaven J (2002) Bilateral Lemierre's syndrome: a case report and literature review. Ear Nose Throat J 81(4):234-236, 238-242.

31. Woywodt A, Merkel S, Buth W, Haller H, Schwarz A (2002) A swollen neck. Lancet 360(9348):1838. https://doi.org/10.1016/S0140-6736(02)11773-9

32. Younus F, Chua A, Tortora G, Jimenez VE (2002) Lemierre's disease caused by co-infection of Arcanobacterium haemolyticum and Fusobacterium necrophorum: a case report. J Infect 45(2):114-117. https://doi.org/10.1053/jinf.2002.1024

33. Zacharek MA, Malani PN, Chenoweth CE (2002) Clinical problem solving: radiology. Radiology quiz case 2: Lemierre syndrome. Arch Otolaryngol Head Neck Surg 128(5):597-599.

34. Belechri M, Siasiakou S, Tsantes A, Petropoulou D, Tsiodra P, Vagiakou E, Malamou LH (2003) Lemmiere's syndrome following infectious mononucleosis. Eur J Intern Med 14(7):447-448. https://doi.org/10.1016/s0953-6205(03)00140-7

35. Ben-David A, Miskin I, Furst A (2003) Lemierre's syndrome: a rare clinical condition diagnosed, exceptionally, by imaging. Isr Med Assoc J 5(11):831-832.

36. Clarke MG, Kennedy NJ, Kennedy K (2003) Serious consequences of a sore throat. Ann R Coll Surg Engl 85(4):242-244. https://doi.org/10.1308/003588403766274935

37. Dalamaga M, Karmaniolas K, Chavelas C, Liatis S, Matekovits H, Migdalis I (2003) Fusobacterium necrophorum septicemia following Epstein-Barr virus infectious mononucleosis. Anaerobe 9(6):285-287. https://doi.org/10.1016/j.anaerobe.2003.08.002

38. Leinung M, Averbeck T, Stöver T (2003) Der interessante Fall Nr. 57 [The interesting case - case no. 57: Lemierre-syndrome following oropharyngeal infection]. Laryngorhinootologie 82(8):578-580. https://doi.org/10.1055/s-2003-41240

39. Ma M, Jauch EC, Johnson MC (2003) A case of Lemierre's syndrome. Eur J Emerg Med 10(2):139-142. https://doi.org/10.1097/00063110-200306000-00015

40. Pulcini C, Vandenbos F, Roth S, Mondain-Miton V, Bernard E, Roger PM, De Salvador-Guillouet F, Hyvernat H, Girard-Pipau F, Mattéi M, Dellamonica P (2003) Syndrome de Lemierre: à propos de 6 cas [Lemierre's syndrome: a report of six cases]. Rev Med Interne 24(1):17-23. https://doi.org/10.1016/s0248-8663(02)00717-8

41. Ramirez S, Hild TG, Rudolph CN, Sty JR, Kehl SC, Havens P, Henrickson K, Chusid MJ (2003) Increased diagnosis of Lemierre syndrome and other Fusobacterium necrophorum infections at a Children's Hospital. Pediatrics 112(5):380. https://doi.org/10.1542/peds.112.5.e380

42. Valla F, Berchiche C, Floret D (2003) Nécrobacillose et syndrome de Lemierre: à propos d'un cas [Necrobacillosis and Lemierre's syndrome: case report]. Arch Pédiatr 10(12):1068-1070. https://doi.org/10.1016/s0929-693x(03)00414-7

43. Williams MD, Kerber CA, Tergin HF (2003) Unusual presentation of Lemierre's syndrome due to Fusobacterium nucleatum. J Clin Microbiol 41(7):3445-3448. https://doi.org/10.1128/JCM.41.7.3445-3448.2003

44. Busko JM, Triner W. (2004) Lemierre syndrome in a child with recent pharyngitis. CJEM.;6(4):285-287. https://doi.org/10.1017/s1481803500009283

45. Haasper C, Tecklenburg FW, Cochran JB, Habib DM, Smith CD. (2004) A pain in the neck can lead to pain in the belly: Lemierre’s syndrome. Int Pediatr 19:185–187.

46. Kara E, Sakarya A, Keleş C, Borand H, Pekindil G, Göktan C (2004) Case of Lemierre's syndrome presenting with thyroid abscess. Eur J Clin Microbiol Infect Dis 23(7):570-572. https://doi.org/10.1007/s10096-004-1161-1

47. Ajulo P, Qayyum A, Brewis C, Innes A (2005) Lemierre's syndrome: the link between a simple sore throat, sore neck and pleuritic chest pain. Ann R Coll Surg Engl 87(4):303-305. https://doi.org/10.1308/1478708051757

48. Aliyu SH, Yong PF, Newport MJ, Zhang H, Marriott RK, Curran MD, Ludlam H (2005) Molecular diagnosis of Fusobacterium necrophorum infection (Lemierre's syndrome). Eur J Clin Microbiol Infect Dis 24(3):226-229. https://doi.org/10.1007/s10096-005-1298-6

49. Boz GA, Iskender S, Caylan R, Aydin K, Koksal I. (2005) A case of Lemierre's syndrome following Epstein-Barr virus infection. Anaerobe;11(3):185-187. https://doi.org/10.1016/j.anaerobe.2004.12.003

50. Charles K, Flinn WR, Neschis DG (2005) Lemierre's syndrome: a potentially fatal complication that may require vascular surgical intervention. J Vasc Surg 42(5):10231025. https://doi.org/10.1016/j.jvs.2005.07.005

51. Duong M, Wenger J (2005) Lemierre syndrome. Pediatr Emerg Care 21(9):589-593. https://doi.org/10.1097/01.pec.0000177198.91278.3e

52. Nadkarni MD, Verchick J, O'Neill JC (2005) Lemierre syndrome. J Emerg Med 8(3):297-299. https://doi.org/10.1016/j.jemermed.2004.09.011

53. Ochoa R, Goldstein J, Rubin R (2005) Clinicopathological conference: Lemierre's syndrome. Acad Emerg Med 12(2):152-157. https://doi.org/10.1197/j.aem.2004.10.001

54. Rivero Marcotegui M, Sánchez Rodríguez C, Cabal Soto S, Aizcorbe Garralda M (2005) Síndrome de Lemierre y Mycoplasma pneumoniae [Lemierre's syndrome and Mycoplasma pneumoniae]. An Med Interna 22(11):541-543. https://doi.org/10.4321/s0212-71992005001100010

55. Schmid T, Miskin H, Schlesinger Y, Argaman Z, Kleid D (2005) Respiratory failure and hypercoagulability in a toddler with Lemierre's syndrome. Pediatrics 115(5):620-622. https://doi.org/10.1542/peds.2004-2505

56. Huits RM, van Assen S, Wildeboer-Veloo AC, Verschuuren EA, Koeter GH (2006) Prevotella bivia necrobacillosis following infectious mononucleosis. J Infect 53(2):59-63. https://doi.org/10.1016/j.jinf.2005.10.016

57. Jones C, Siva TM, Seymour FK, O'Reilly BJ (2006) Lemierre's syndrome presenting with peritonsillar abscess and VIth cranial nerve palsy. J Laryngol Otol 120(6):502-504 https://doi.org/10.1017/S002221510600034X.

58. Matten EC, Grecu L (2006) Unilateral empyema as a complication of infectious mononucleosis: a pathogenic variant of Lemierre's syndrome. J Clin Microbiol 44(2):659-661. https://doi.org/10.1128/JCM.44.2.659-661.2006

59. Ravn T, Huniche B, Breum L, Jørgen Christensen J (2006) Lemierre's syndrome: still an important clinical entity. Scand J Infect Dis 38(4):299-301. https://doi.org/10.1080/0036554050034900060.

60. Boga C, Ozdogu H, Diri B, Oguzkurt L, Asma S, Yeral M (2007) Lemierre syndrome variant: Staphylococcus aureus associated with thrombosis of both the right internal jugular vein and the splenic vein after the exploration of a river cave. J Thromb Thrombolysis 23(2):151-154. https://doi.org/10.1007/s11239-006-9050-3

61. Cholette JM, Caserta M, Hardy D, Connolly HV (2007) Outcome of pulmonary function in Lemierre's disease-associated acute respiratory distress syndrome. Pediatr Pulmonol 42(4):389-392. https://doi.org/10.1002/ppul.20573

62. Healy B, Llewelyn M, Cavalle F, Bernard M (2007) Lemierre's syndrome in association with a cholesteatoma. Br J Hosp Med (Lond) 68(6):330-331. https://doi.org/10.12968/hmed.2007.68.6.23579

63. Jankowich M, El-Sameed YA, Abu-Hijleh M (2007) A 21-year-old man with fever and sore throat rapidly progressive to hemoptysis and respiratory failure. Diagnosis: Lemierre syndrome with Fusobacterium necrophorum sepsis. Chest 132(5):1706-1709. https://doi.org/10.1378/chest.07-0631

64. Waterman JA, Balbi HJ, Vaysman D, Ayres RA, Caronia CG. (2007) Lemierre syndrome: a case report. Pediatr Emerg Care 23(2):103-105. https://doi.org/10.1097/PEC.0b013e3180302c0f

65. Westhout F, Hasso A, Jalili M, Afghani B, Armstrong W, Nwagwu C, Ackerman LL (2007) Lemierre syndrome complicated by cavernous sinus thrombosis, the development of subdural empyemas, and internal carotid artery narrowing without cerebral infarction. Case report. J Neurosurg 106(1 Suppl):53-56. https://doi.org/10.3171/ped.2007.106.1.53

66. Garimorth K, Kountchev J, Bellmann R, Semenitz B, Weiss G, Joannidis M (2008) Lemierre's syndrome following infectious mononucleosis. Wien Klin Wochenschr 120(5-6):181-183. https://doi.org/10.1007/s00508-008-0937-1

67. Georgopoulos S, Korres S, Riga M, Balatsouras D, Kotsis G, Ferekidis E (2008) Lemierre's syndrome associated with consumption coagulopathy and acute renal failure: a case report. J Laryngol Otol 122(5):527-530. https://doi.org/10.1017/S0022215107007256

68. Hot A, Coppere B, Ninet J, Thiebault A (2008) Lemierre syndrome caused by Leptotrichia buccalis in a neutropenic patient. Int J Infect Dis 12(3):339-340. https://doi.org/10.1016/j.ijid.2007.08.009

69. Passalidou P, Berlioz M, Bailly C, Boutté P (2008) Syndrome de Lemierre: une infection oropharyngée compliquée [Lemierre syndrome: a complication of an oropharyngeal infection]. Arch Pédiatr 15(12):1775-1778. https://doi.org/10.1016/j.arcped.2008.09.009

70. van Delft E, Vandewall M, Curiel FB, Rutten MJ, Schneeberger PM (2008) A previously healthy 15-year-old girl with high fever and progressive dyspnoea. Eur J Pediatr 167(6):711-713. https://doi.org/10.1007/s00431-007-0628-3

71. van Dijk EJ, van Swieten JC, Koudstaal PJ (2008) Meningitis, cranial nerve palsies and bilateral cerebral infarcts: a neurological variant of Lemierre's syndrome. J Neurol 255(10):1588-1589. https://doi.org/10.1007/s00415-008-0965-9

72. Fernández-Suárez A, Aguilar Benítez JM, López Vidal AM, Díaz Iglesias JM (2009) Lemierre's syndrome and septicaemia caused solely by Arcanobacterium haemolyticum in a young immunocompetent patient. J Med Microbiol 58(Pt 12):1645-1648. https://doi.org/10.1099/jmm.0.007492-0

73. Gashau A (2009) Lemierre syndrome: a needle in a haystack. BMJ Case Rep 2009:bcr12.2008.1394. https://doi.org/10.1136/bcr.12.2008.1394

74. Kushawaha A, Popalzai M, El-Charabaty E, Mobarakai N (2009) Lemierre's syndrome, reemergence of a forgotten disease: a case report. Cases J 2:6397. https://doi.org/10.1186/1757-1626-0002-0000006397

75. Lu MD, Vasavada Z, Tanner C (2009) Lemierre syndrome following oropharyngeal infection: a case series. J Am Board Fam Med 22(1):79-83. https://doi.org/10.3122/jabfm.2009.01.070247

76. Sarjomaa M, Liyanarachi KV, Brekke H (2009) Fra tonsillitt til koksitt [From tonsillitis to coxitis]. Tidsskr Nor Laegeforen 129(17):1754-1755. https://doi.org/10.4045/tidsskr.08.0218

77. Bonhoeffer J, Trachsel D, Hammer J, Nava E, Heininger U (2010) Lemierre syndrome and nosocomial transmission of Fusobacterium necrophorum from patient to physician. Klin Pädiatr 222(7):464-466. https://doi.org/10.1055/s-0030-1263144

78. Chacko EM, Krilov LR, Patten W, Lee PJ (2010) Lemierre's and Lemierre's-like syndromes in association with infectious mononucleosis. J Laryngol Otol 124(12):1257-1262. https://doi.org/ 10.1017/S0022215110001568

79. Dirks J, Bowie D (2010) Sore throat progressing to embolic sepsis: a case of Lemierre's syndrome. Can Respir J 17(1):20-22. https://doi.org/10.1155/2010/950709

80. Lundblom K, Jung K, Kalin M (2010) Lemierre syndrome caused by co-infection by Arcanobacterium haemolyticum and Fusobacterium necrophorum. Infection 38(5):427-429. https://doi.org/10.1007/s15010-010-0046-8

81. Malhotra A, Westesson PL (2010) Lemierre syndrome. Pediatr Radiol 40(8):1451. https://doi.org/10.1007/s00247-009-1442-z

82. Peer Mohamed B, Carr L (2010) Neurological complications in two children with Lemierre syndrome. Dev Med Child Neurol 52(8):779-781. https://doi.org/10.1111/j.1469-8749.2010.03718.x

83. Ridgway JM, Parikh DA, Wright R, Holden P, Armstrong W, Camilon F, Wong BJ (2010) Lemierre syndrome: a pediatric case series and review of literature. Am J Otolaryngol 31(1):38-45. https://doi.org/10.1016/j.amjoto.2008.09.006

84. Tran D, Maradia K, Montero J (2010) Lemierre syndrome: a rare disease affecting previously healthy, young individuals. South Med J 103(11):1190. https://doi.org/10.1097/SMJ.0b013e3181f1edb7

85. Vaid A, Kornfeld M (2010) Unforgettable? J Hosp Med 5(8):486-490. https://doi.org/10.1002/jhm.815

86. Vargiami EG, Farmaki E, Tasiopoulou D, Zafeiriou DI, Badouraki M, Anastasiou A, Karkos C, Gombakis N, Athanasiou-Metaxa M (2010) A patient with Lemierre syndrome. Eur J Pediatr 169(4):491-493. https://doi.org/10.1007/s00431-009-1134-6

87. Yamaguchi M, Nishizawa H, Yasumoto T, Kimura T, Takeuchi Y, Takeshita A, Takase T, Fujimoto H, Nakano M, Fukunaga M (2010) Lemierre syndrome with blepharoptosis. Intern Med 49(8):753-757. https://doi.org/10.2169/internalmedicine.49.2779

88. Chanin JM, Marcos LA, Thompson BM, Yusen RD, Dunne WM Jr, Warren DK, Santos CA (2011) Methicillin-resistant Staphylococcus aureus USA300 clone as a cause of Lemierre's syndrome. J Clin Microbiol 49(5):2063-2066. https://doi.org/10.1128/JCM.02507-10

89. Mewawalla P, Bauer F, Dasanu CA. (2011) Lemierre syndrome caused by group C streptococci and associated with red cell aplasia and immune thrombocytopenia. South Med J 104(3):244-246. https://doi.org/10.1097/SMJ.0b013e31820604b7

90. Kraus CL, Culican SM (2012) Challenging presentations of cavernous sinus thrombophlebitis. J Ophthalmic Inflamm Infect 2:133-136. https://doi.org/10.1007/s12348-011-0053-7

91. Kuppalli K, Livorsi D, Talati NJ, Osborn M (2012) Lemierre's syndrome due to Fusobacterium necrophorum. Lancet Infect Dis 12(10):808-815. https://doi.org/10.1016/S1473-3099(12)70089-0

92. Miller B, Khalifa Y, Feldon SE, Friedman DI. (2012) Lemierre syndrome causing bilateral cavernous sinus thrombosis. J Neuroophthalmol 32(4):341-344. https://doi.org/10.1097/WNO.0b013e31825e42ae.

93. Paul SP, Beri R, Linney MJ (2012) Lemierre's syndrome: a sinister sore throat every clinician should remember. Turk J Pediatr 54(5):528-531.

94. Hawes D, Linney MJ, Wilkinson R, Paul SP (2013) Lemierre's syndrome: the importance of early detection. Br J Nurs 22(18):1075-1078. https://doi.org/10.12968/bjon.2013.22.18.1075

95. Johannesen K, Bødtger U, Heltberg O (2014) Lemierre's syndrome: the forgotten disease. J Thromb Thrombolysis 37(3):246-248. https://doi.org/10.1007/s11239-013-0931-y

96. Klein NC, Petelin A, Cunha BA (2013) Mycoplasma pneumoniae preceding Lemierre's syndrome due to Fusobacterium nucleatum complicated by acute Epstein-Barr virus (EBV) infectious mononucleosis in an immunocompetent host. Heart Lung 42(1):74-76. https://doi.org/10.1016/j.hrtlng.2012.02.015

97. Botros J, Rencic J, Centor RM, Henderson MC (2014) Anchors away. J Gen Intern Med 29(10):1414-1418. https://doi.org/10.1007/s11606-014-2879-9

98. Gunatilake SS, Yapa LG, Gallala M, Gamlath R, Rodrigo C, Wimalaratna H (2014) Lemierre's syndrome secondary to community-acquired methicillin-resistant Staphylococcus aureus infection presenting with cardiac tamponade, a rare disease with a life-threatening presentation: a case report. Int J Emerg Med 7:39. https://doi.org/10.1186/s12245-014-0039-y

99. Olson KR, Freitag SK, Johnson JM, Branda JA. (2014) Case records of the Massachusetts General Hospital. Case 36-2014. An 18-year-old woman with fever, pharyngitis, and double vision. N Engl J Med 371(21):2018-2027. https://doi.org/10.1056/NEJMcpc1310001

100. Hadjinicolaou AV, Philippou Y (2015) Lemierre's syndrome: a neglected disease with classical features. Case Rep Med 2015:846715. https://doi.org/10.1155/2015/846715

101. Wong AP, Duggins ML, Neil T (2015) Internal jugular vein septic thrombophlebitis (Lemierre syndrome) as a complication of pharyngitis. J Am Board Fam Med 28(3):425-430. https://doi.org/10.3122/jabfm.2015.03.140131

102. Alfreijat M (2016) A case of Lemierre's Syndrome with a brief literature review. J Infect Public Health 9(5):681-683. https://doi.org/10.1016/j.jiph.2016.01.006

103. Faraone A, Fortini A, Nenci G, Boccadori C, Mangani V, Oggioni R (2016) Fusobacterium necrophorum pharyngitis complicated by Lemierre's syndrome. Case Rep Med 2016:3608346. https://doi.org/10.1155/2016/3608346.

104. Harper LK, Pflug K, Raggio B, April D, Milburn JM (2016) Clinical images: Lemierre Syndrome: the forgotten disease? Ochsner J 16(1):7-9.

105. Bahall M, Giddings S, Bahall K (2017) Lemierre's syndrome: forgotten, but not absent. BMJ Case Rep 2017:bcr2017221203. https://doi.org/10.1136/bcr-2017-221203

106. Kumral AVW, Petersen WC Jr, Heitz C, Waggoner-Fountain LA, Belyea BC (2017) Lemierre's syndrome as a trigger for secondary hemophagocytic lymphohistiocytosis. J Pediatr Hematol Oncol 39(6):325-327. https://doi.org/10.1097/MPH.0000000000000755

107. Meade CM, Cantos VD, Nasri H, Serbanescu M, Anderson EJ (2017) Epidural abscess in Lemierre׳s syndrome. Am J Med Sci 354(3):325-326. https://doi.org/10.1016/j.amjms.2016.11.017

108. Rae J, Misselbrook K (2017) Lemierre's syndrome - a rare cause of disseminated sepsis requiring multi-organ support. J Intensive Care Soc 18(4):329-333. https://doi.org/10.1177/1751143717698978

Rana MA, Kumar Y, Lashari AA, Mady AF (2017) human infection with fusobacterium necrophorum without jugular venous thrombosis: a varied presentation of Lemierre's syndrome. Case Rep Infect Dis 2017:5358095. https://doi.org/10.1155/2017/5358095

109. Hedenmark J, Holm K, Moberger F, Torell E (2018) Lemierres syndrom – en påminnelse om »den glömda sjukdomen« - God kunskap under hela vårdkedjan viktigt för att i tid väcka misstanke. Lakartidningen 115:EWPE.

110. Mangan J, Shah D, Troy A, Dawgert D (2018) Case 2: jaw pain, pain on deep inspiration, and severe odynophagia in an 18-year-old boy. Pediatr Rev 39(4):211-212. https://doi.org/10.1542/pir.2015-0123

111. Murray A, Rath T, Wördehoff L, Schuler-Lüttmann S, Baumgärtel MW (2018) Dysphagie und Angina tonsillaris bei einem 25-jährigen Patienten: die fast vergessene schwere Komplikation einer häufigen Krankheit [Dysphagia and tonsillitis in a 25-year-old male patient: the nearly forgotten severe complication of a common ailment]. Internist (Berl) 59(5):494-496. https://doi.org/10.1007/s00108-017-0364-3

113. Stubington TJ, James P (2018) Lemierre's syndrome: a pain in the neck with far-reaching consequences. BMJ Case Rep 2018:bcr2017222723. https://doi.org/10.1136/bcr-2017-222723

114. Alves S, Stella L, Carvalho I, Moreira D (2019) Lemierre's syndrome: a disguised threat. BMJ Case Rep 12(4):228397. https://doi.org/10.1136/bcr-2018-228397

115. Dasari SP, Gill H, Bodette H, Brandes E, Jha P (2020) A Challenging case of Lemierre's syndrome with central nervous system involvement and a comprehensive review. Cureus 12(8):10131. https://doi.org/10.7759/cureus.10131

116. Gusnowski EM, Morrison LJ, Bois AJ (2020) Infectious brachial plexopathy and septic arthritis of the shoulder due to Lemierre's syndrome: a case report and literature review. JBJS Case Connect 10(4):20.00362. https://doi.org/10.2106/JBJS.CC.20.00362

117. Karn MN, Johnson NP, Yaeger SK, Fugok KL (2020) A teenager with fever, chest pain, and respiratory distress during the coronavirus disease 2019 pandemic: a lesson on anchoring bias. J Am Coll Emerg Physicians Open 1(6):1392-1394. https://doi.org/10.1002/emp2.12261

118. Sattar Y, Susheela AT, Karki B, Liaqat A, Ullah W, Zafrullah F (2020) Diagnosis and management of Lemierre's syndrome presented with multifocal pneumonia and cerebral venous sinus thrombosis. Case Rep Infect Dis 2020:6396274. https://doi.org/10.1155/2020/6396274

119. Barguil S, Sousa Alves A, Christen JR, De Jerphanion C, Valero J, Gaillard T, Karkowski L (2021) Pharyngeal abscess complicated by Lemierre's syndrome: identification of an atypical germ. IDCases 25:01180. https://doi.org/10.1016/j.idcr.2021.e01180

120. Costa F, Matos Bela M, Ferreira I, Cidade Rodrigues C, América Silva A (2021) Not all pulmonary densifications are COVID-19: a case report about Lemierre's syndrome. Cureus 13(6):15984. https://doi.org/10.7759/cureus.15984

121. Jaber TM, Saini V, Ogbebor O, Dumont T, Balaan M, Lega M, Cheema T (2021) Lemierre's syndrome: a case series. Cureus 13(10):18436. https://doi.org/10.7759/cureus.18436

122. Litt MJ, Gaffney R, Vaidya A, Montgomery MW (2021) Hard to swallow. N Engl J Med 385(14):47. https://doi.org/10.1056/NEJMimc2103214

123. Maier-Stocker C, Hellwig D, Hanses F (2021) Lemierre's syndrome following infectious mononucleosis: an unusual reason for neck pain. Lancet Infect Dis 21(7):1050. https://doi.org/10.1016/S1473-3099(21)00137-7

124. Marques TM, Almeida A, Cruz L, Ferreira T (2021) Rapidly progressing incomplete Lemierre syndrome. J Postgrad Med.;67(4):249-250. https://doi.org/10.4103/jpgm.JPGM_45_21

125. Mohiuddin Z, Manes T, Emerson A (2021) Fusobacterium necrophorum bacteremia with evidence of cavitary pulmonary lesion. Cureus 13(11):19537. https://doi.org/10.7759/cureus.19537

126. Moretti M, De Geyter D, Goethal L, Allard SD (2021) Lemierre's syndrome in adulthood, a case report and systematic review. Acta Clin Belg 76(4):324-334. https://doi.org/10.1080/17843286.2020.1731661

127. Vera Nieves B, Lindblad G, Gupta J, Hughes J, Rivero A (2021) An atypical presentation of Lemierre's syndrome: complicated by thrombotic thrombocytopenic purpura. Cureus 13(1):12728. https://doi.org/10.7759/cureus.12728

128. Nguyen HD, Whitley-Williams PN, Uppaluri LP, Sangani J, Simon ML, Baig AS (2022) Case report of atypical Lemierre's Syndrome associated with Fusobacterium nucleatum infection without internal or external jugular venous thrombophlebitis. Respir Med Case Rep 37:101651. https://doi.org/10.1016/j.rmcr.2022.101651

129. Ueno K, Horiuchi H, Utada S, Shinomiya Y, Sogo A, Miyagawa T, Niida S, Okano H, Suzuki N, Otsuka T, Miyazaki H, Furuya R (2022) Lemierre's syndrome as a sexually transmitted disease due to Porphyromonas asaccharolytica suspected to be caused by pharyngitis due to Mycoplasma pneumoniae and Epstein-Barr virus. Cureus 14(8):28219. https://doi.org/10.7759/cureus.28219

130. Au PH, Nwabara K, Gvazava N, Ejiofor S, Ghous G (2023) Lemierre syndrome: a diagnosis behind the veil. Case Rep Infect Dis 2023:2273954. https://doi.org/10.1155/2023/2273954

131. Bourgeaud J, Delabays B, Van den Bogaart L, Ratano D. Complex (2023) Lemierre syndrome with multisystemic abscesses. BMJ Case Rep 16(4):254638. https://doi.org/10.1136/bcr-2023-254638.

132. Zhang Y, Zeng Y (2023) Lemierre's syndrome under the disguise of COVID-19 pneumonia: a case report and systematic review. Cureus 15(9):45827. https://doi.org/10.7759/cureus.45827
